# Smoking and alcohol drinking and risk of non-union or delayed union after fractures: A protocol for systematic review and dose–response meta-analysis

**DOI:** 10.1097/MD.0000000000018744

**Published:** 2020-01-31

**Authors:** Bin Xu, Lingxiao Chen, Jae Hyup Lee

**Affiliations:** a Department of Orthopedic Surgery, College of Medicine, Seoul National University, Seoul, Republic of Korea; b Department of Orthopedic Surgery, Tianjin Hospital, Tianjin University, Tianjin, China; c Institute of Bone and Joint Research, Kolling Institute, Sydney Medical School, Faculty of Medicine and Health, University of Sydney, Sydney, New South Wales, Australia; d Department of Orthopedic Surgery, SMG-SNU Boramae Medical Center; e Institute of Medical and Biological Engineering, Medical Research Center, Seoul National University, Medical Research Center, Seoul, Republic of Korea.

**Keywords:** alcohol drinking, dose–response, fracture healing, smoking, systematic review

## Abstract

**Introduction:**

To the best of our knowledge, there is no consensus on dose–response between smoking, alcohol drinking, and bone healing. The aim of the present study is to conduct a comprehensive systematic review and dose–response meta-analysis of studies to estimate the influence of smoking and alcohol use on the success of non-pathologic bone fracture healing in adult patients.

**Methods:**

A systematic search will be performed using MEDLINE, EMBASE and Cochrane CENTRAL, CINAHL, and AMED databases to identify randomized controlled trials and observational studies which have assessed the effect of smoking or alcohol drinking on fracture healing. Primary outcomes include delayed union or nonunion rate and time to union. Secondary outcomes are common complications which occur during bone healing including malunion and wound infection. Risk of bias will be evaluated using the Quality In Prognosis Studies (QUIPS) tool for quality assessment of each study. Dose–response meta-analysis will be performed between smoking, alcohol drinking, and bone healing. Evaluation of the quality of evidence will be conducted using the Grading of Recommendations Assessment, Development, and Evaluation (GRADE) system.

**Results:**

The present study will assess the effects of smoking and alcohol drinking on non-pathologic bone fracture healing in adult patients.

**Conclusion:**

We hope that this systematic review and dose–response meta-analysis will provide high quality evidence on dose–response between smoking, alcohol drinking, and bone fracture healing.

**PROSPERO registration number:**

CRD42019131454.

## 1 Introduction

The annual occurrence rate of fractures in the whole skeletal system is 3.6 per 100 people in England.^[1]^ Although most of the fractures heal eventually, it has been reported that about 0.1 million of 2 million long bone fractures are converted into nonunion annually in USA.^[2]^ Delayed union and nonunion of fractures not only extend the length of hospital stay but also increase economic burden of family.^[3–7]^

The main clinical factors related to inhibition of bone fracture healing include smoking and alcohol consumption.^[8,9]^ Previous studies have shown that smokers have higher risk for worse mechanical characteristics of intrinsic trabecular bone,^[10]^ bone fractures,^[11,12]^ delayed union and non-union after open or closed fractures,^[13–15]^ postoperative fracture healing complications including surgical site infection,^[14,16,17]^ and mortality after fracture.^[18,19]^ Numerous factors including reduction in peripheral blood flow caused by nicotine, which is a power vasoconstrictor, reduction in the oxygen-carrying capacity of hemoglobin due to bonding of hemoglobin with carbon monoxide, and obstacles in aerobic metabolism through inhibition of cytochrome c oxidase caused by hydrogen cyanide are the negative effects of smoking on bone healing.^[13]^ Exposure to high dose of nicotine has been reported to inhibit bone regeneration in a rabbit osteotomy and distraction model.^[20]^ Increase in nonunion rate after plating in proximal humeral fractures along with smoking level was also found in a clinical study.^[21]^ Thus, smoking level is considered as an important factor to affect bone healing.

Regarding alcohol drinking, published studies indicate that alcohol consumption has harmful effects on bone health including a higher rate of fracture^[22–25]^ and higher incidence of infection at the surgical site.^[26]^ Decrease in integrin β1 receptor and osteopontin (OPN) expression is considered as the possible mechanism of delayed union and nonunion of fractures caused by alcohol. Interaction between OPN and integrin β1 receptor induces mesenchymal stem cells (MSC) migration to fracture site and endochondral ossification.^[27,28]^ Alcohol consumption has been reported to affect bone repair in a rat fibula osteotomy model in a dose-dependent manner.^[8]^ Consequently, alcohol consumption is considered as an important factor related to bone healing.

Only one meta-analysis has been published with a focus on the effect of smoking on the outcomes of fracture healing.^[13]^ However, the limitations of the study were as follows:

1. absence of dose–response analysis and
2. the search date of the meta-analysis that was May 2015.

In addition, there exists no meta-analysis about the effect of alcohol drinking on fracture healing. Therefore, our aim is to comprehensively perform a meta-analysis that will investigate dose–response among smoking or alcohol drinking and fracture healing in the entire skeletal system. The results of this meta-analysis might identify prognostic factors associated with bone healing which could help develop the impact of prognostic models for individualized prediction of fracture healing outcomes.

The present study presents the protocol for a systematic review and dose–response meta-analysis, which will compare delayed union or nonunion rates, time to union and complications related to bone healing between smokers and non-smokers and between alcohol users and non-alcohol users. Subsequently, the quantitative relationship between extent of smoking and alcohol consumption and the outcomes mentioned above will be explored.

## 2 Objective

The objective of the current systematic review and dose–response meta-analysis is to investigate the influence of smoking or alcohol use on the success of bone healing in adult patients after non-pathologic fractures by answering the following question: What is the influence of smoking and alcohol drinking on the healing rate, healing time, and complication rate including malunion and wound infection.

## 3 Methods

The current protocol is presented following the Preferred Reporting Items for Systematic Review and Meta-Analysis Protocols (PRISMA-P) guidance^[29]^ and the reporting of the meta-analysis will follow the guidelines proposed by Meta-analysis Of Observational Studies in Epidemiology (MOOSE).^[30]^ This protocol has been registered in the international prospective register of systematic reviews (PROSPERO; Registration number CRD42019131454).

### 3.1 Eligibility criteria

#### 3.1.1 Study designs

Randomized controlled trials (RCTs) and observational studies (cohort, case–control, and cross-sectional studies) will be included in the study.

#### 3.1.2 Participants

We will include studies examining adults aged over 18 years who had traumatic bone fractures in any location. Patients with fractures caused by pathological factors, such as cancer, kidney disease, human immunodeficiency virus, and patients who underwent joint replacement using prosthesis or amputation for first-time treatment will be excluded based on the reason of slow healing and low healing rate. If mixed participants with traumatic fractures and pathological fractures exist in a study, we will include the study in which patients with pathological fractures account for <20%.^[31]^

#### 3.1.3 Exposure

The studies exploring the effect of current smoking and alcohol consumption on bone healing in patients with traumatic fractures will be included. Definitions of current smoking and current alcohol consumption are shown in the data items section.

#### 3.1.4 Comparators

Patients who have never smoked and who never drank will be the comparators of this study. Definitions of never smokers and never drinkers are also presented in the data items section.

#### 3.1.5 Outcomes

The outcomes will comprise of delayed union or nonunion rate, time to union, malunion rate, and wound infection rate.

### 3.2 Information sources

We will undertake a systematical search using MEDLINE, EMBASE and Cochrane CENTRAL, CINAHL and AMED databases from inception with language restriction of English. Also, the manual search will be performed by screening references of included studies for relevant references.

### 3.3 Search strategy

Search strategies will be performed by an experienced librarian using keywords of “smoking,” “nicotine,” “cigarettes,” “alcohol,” “alcohol drinking,” “alcoholism,” “fracture,” “bone healing,” “delayed union,” “nonunion,” “malunion,” and “surgical wound infection” through MEDLINE, EMBASE and Cochrane CENTRAL, CINAHL and AMED databases with the limitation to English. The full search strategy used in MEDLINE via OVID is presented in Table 1.

**Table 1:**
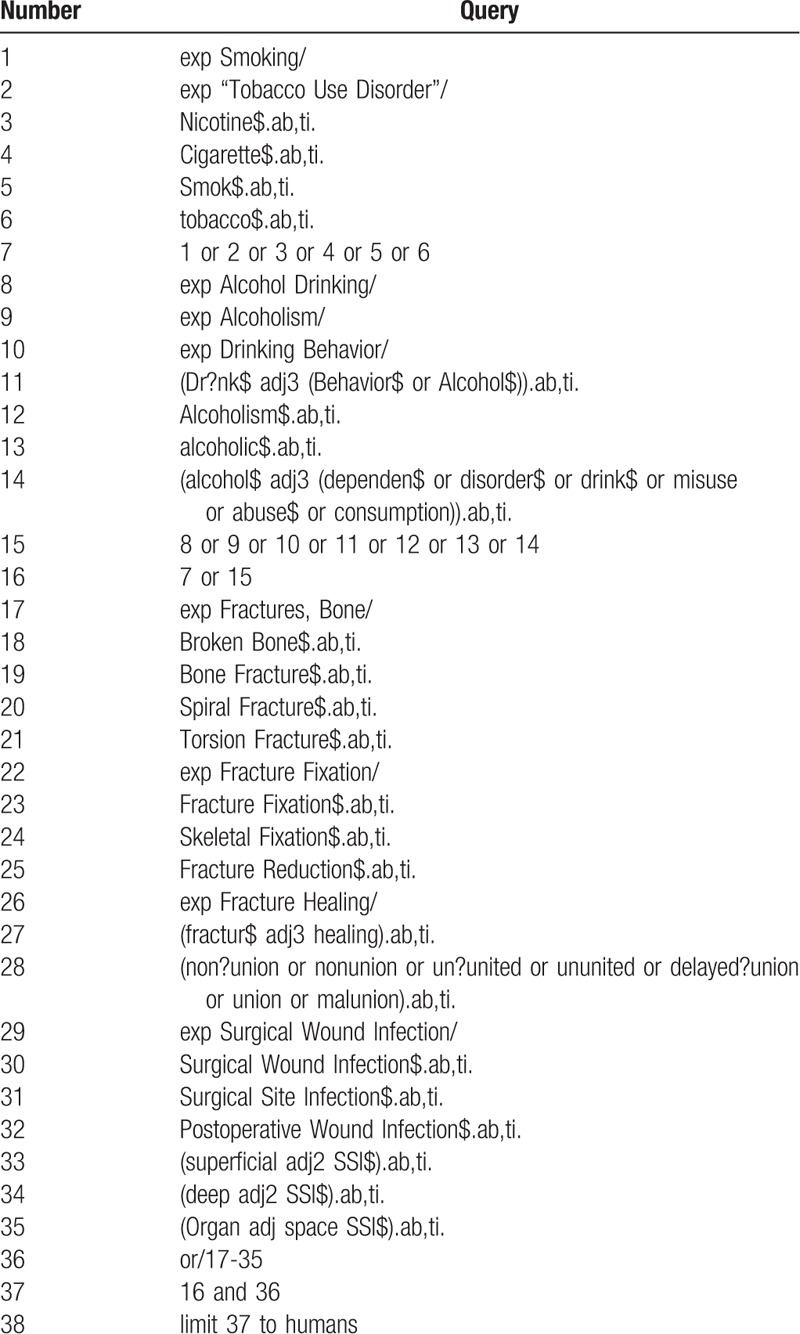
MEDLINE via OVID search strategy.

### 3.4 Study records

#### 3.4.1 Data management

We will use EndNote X7 to manage search results, remove duplicate literatures, and select studies based on the eligibility criteria.

#### 3.4.2 Selection process

**Figure 1:**
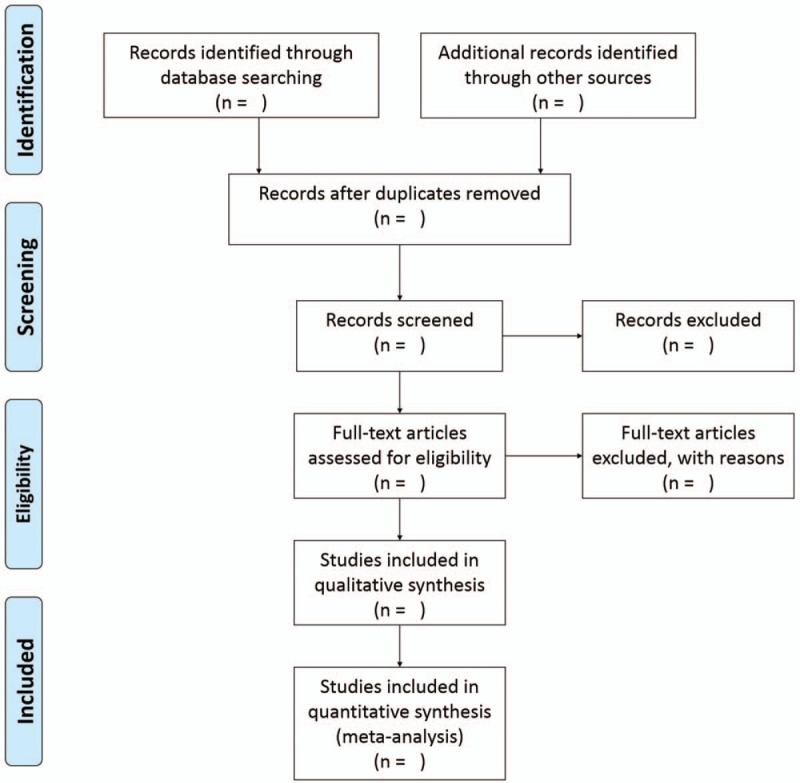
PRISMA flow diagram.

The selection process will be presented in a PRISMA-compliant flow chart (Fig. 1). Study selection will be independently performed by two authors by screening the titles and abstracts of literatures. Moreover, further selection will be done by reading full text of records identified potentially eligible studies. Any disagreement generated during selection process will be resolved based on mutual discussion with each other or with the help from the third reviewer. In addition, the information about the excluded studies whose full texts will be read along with the reasons for exclusion would be provided.

#### 3.4.3 Data collection process

Two authors will independently perform data extraction from eligible studies using a standardized form. Disagreement will be resolved based on mutual discussion with each other or by the third reviewer. In the case of uncertainties, we will contact the original investigators for resolving the issue.

#### 3.4.4 Data items

The data that will be extracted are:

1. basic characteristics of the studies (first author, journal name, publication year, group, study design, etc);
2. characteristics of the participants (sex, age, race, BMI, height, weight, and fracture location, etc);
3. other factors that may slow bone healing time (diabetes, use of nonsteroidal anti-inflammatory drugs [NSAIDs], and fluoroquinolone family of antibiotics)^[32]^;
4. exposure details (treatment, smoking type [e.g., cigarette, cigar, and waterpipe], smoking dose, alcohol type [e.g., beer, wine, and spirits], and level of alcohol consumption); and
5. outcome measures (sample sizes, delayed union and nonunion rate, time to union, and complications during bone healing, etc).

Current smokers are defined as participants who smoke at least 10 cigarettes per day for the past 1 year and never smokers are participants who did not smoke during the past year and smoked <100 cigarettes during their lifetime.^[33]^ Alcohol intake will be converted into grams per day based on the standard drink size (1 mL = 0.8 g, 1 oz = 28.35 g, 1 drink = 14 g, and 1 unit = 7.9 g). And level of alcohol drinking will be classified into light (<7 g/day), moderate (7–14 g/day), heavy (>14 g/day), and binge drinking (28–35 g/day).^[34]^ Never drinker is defined as participants with no alcohol consumption within the preceding 12 months.^[35]^ Current drinker is defined as participants who drank alcohol within the past 12 months.

Regarding missing data of eligible studies, we will contact the original authors. Also, reminder emails will be sent twice at most within a period of 8 weeks and will wait for the next 12 weeks at the maximum for the reply.

### 3.5 Outcomes and prioritization

The primary outcomes comprise of delayed union or nonunion rate and time to union. Delayed union and nonunion are diagnosed when the time frame for fracture union is from 3 to 6 months and generally over 9 months, respectively, which varies based on fracture location and the level of soft-tissue related injury.^[2]^ Time to union is defined as the time from initiation of bone fracture to the occurrence of clinical union (stabilization of fracture site and painlessness) and radiographic union (trabecular or cortical bone crosses the fracture site).^[32]^ Secondary outcomes include common complications occurring during bone healing including malunion rate and wound infection rate.^[36]^ Malunion is defined as a fracture that is not anatomically healed. Wound infection is defined as infection, which is probably related to operation, occurring within 30 days after the operation when there is no implant left or within 1 year when there is implant left in surgical site.^[37]^

The hypothesis is that outcomes of RCTs and cohort studies might be similar to case-control and cross-sectional studies.

### 3.6 Methodological quality assessment of individual studies

Two authors will independently evaluate the risk of bias in each included study using the Quality In Prognosis Studies (QUIPS) tool for quality assessment of RCT, cohort study, case-control study, and cross-sectional study. In total, representativeness of the study sample (study participants domain), whether participants with follow-up data represent persons enrolled in the study (study attrition domain), adequacy of prognostic factor measurement (prognostic factor measurement domain), the adequacy of outcome measurement (outcome measurement domain), potential confounding factors (study confounding domain), and the appropriateness of the study's statistical analysis and completeness of reporting (statistical analysis and reporting domain) will be assessed for each study. Studies will be classified into low, moderate, and high risk of bias according to the results. Any controversy during assessment will be managed via discussion or consulting the third author. The results of risk of bias of each study will be shown in a table form.

### 3.7 Assessment of heterogeneity

We will assess statistical heterogeneity between studies by calculating *I*^2^ value by adhering to the Cochrane Handbook for Systematic Reviews of Interventions.^[31]^ Value of *I*^2^ larger than 75% means considerable statistical heterogeneity. Data of studies with low statistical heterogeneity, clinical heterogeneity, and methodological heterogeneity will be used for quantitative synthesis.

### 3.8 Data synthesis

If more than 2 studies eligible are included, as data is expected to vary across studies, meta-analysis will be performed using random-effects model^[38]^ to combine data of bone healing outcomes with the tools of RevMan 5.3 (Denmark) and R software 3.6.1 using package “metafor.” For continuous data including the time to union, mean difference (MD) with 95% confident intervals (CIs) will be used to evaluate effect size. Dichotomous data comprising of delayed and nonunion rate and complications will be analyzed using risk ratios (RRs) with 95% CIs. If studies show substantial clinical/methodological heterogeneity or <2 studies are included, quantitative synthesis will not be performed. A narrative review of results will be illustrated. In addition, dose–response meta-analysis will be conducted using generalized least squares for trend estimation. Estimation of linear trends will be assessed using the correlated natural logs of RR across exposure categories. We will test the non-linear trend through restricted cubic spline model.^[39]^ The median or mean level of the exposure will be allocated to each relevant exposure category. Calculation of the median will be performed using the midpoint of each category if no available data is acquired. Meta-regression will be performed as follows: age; ratio of male to female; BMI; sample size; use of NSAIDs; use of fluoroquinolone family of antibiotics; and diabetes.

#### 3.8.1 Subgroup analysis and investigation of heterogeneity

If the necessary data are available, subgroup analyses will be done comparing RCTs vs. cohort vs. case-control and cross-sectional studies.

For primary outcomes (delayed union or nonunion rate and time to union), sensitivity analyses will be conducted in order to confirm whether our findings are destabilized by factors as follows:

1. studies with low risk of bias and
2. studies performed with no commercial fund assistance.

Results of sensitivity analysis will be presented in a table form.

### 3.9 Publication bias assessment

Publication bias of included studies will be evaluated using funnel plots according to Cochrane Handbook for Systematic Reviews for Interventions^[31]^ if more than 10 studies are included.

### 3.10 Grading quality of evidence

Quality of evidence will be assessed by two authors independently using the Grading of Recommendations Assessment, Development and Evaluation (GRADE) system.^[40]^ Evidence will be categorized as high, moderate, low, and very low according to study design, risk of bias, inconsistency, indirectness, imprecision, publication bias, magnitude of the effect, dose–response gradient, and confounding bias.

### 3.11 Ethics and dissemination

Ethical approval is not required for this study because data to be used is from published literatures. Our findings will be expected to provide important data support for clinical treatment and prevention. The results of our review will be published in a peer-reviewed scientific journal and presented at international conferences.

### 3.12 Patient and public involvement

No patient was involved in this protocol for systematic review and meta-analysis.

## 4 Discussion

Delayed union and nonunion of non-pathologic bone fracture are major public health problems, that not only reduce quality of life of patients but also increase economic burden of family. To date, only one meta-analysis has evaluated association between smoking and fracture healing. However, search date was May 2015. In addition, no meta-analysis has assessed the effect of alcohol drinking on fracture healing. Moreover, dose–response between smoking, alcohol drinking and fracture healing is still not known. We will perform this systematic review and meta-analysis of the effect of smoking, alcohol drinking on delayed union or nonunion, time to union, malunion and wound infection to provide patients and clinicians high quality evidence on association between them.

## Acknowledgments

We thank PARK Eun-Sun at Seoul National university Medical library for her contribution in developing the search strategy in MEDLINE database via Ovid.

## Author contributions

**Conceptualization:** Lingxiao Chen, Jae Hyup Lee.

**Methodology:** Bin Xu, Lingxiao Chen.

**Writing –original draft:** Bin Xu.

**Writing – review & editing:** Bin Xu, Lingxiao Chen, Jae Hyup Lee.

Jae Hyup Lee: 0000-0002-2141-0266.
